# Relapse of Hodgkin's disease revealed by skin involvement

**DOI:** 10.1002/ccr3.6244

**Published:** 2022-09-02

**Authors:** Ouadii Abakarim, Oumaima Maghnouj, Abderrachid Hamdaoui, Fatima Zahra Lahlimi, Illias Tazi

**Affiliations:** ^1^ Department of Clinical Hematology & Bone Marrow Transplantation, University Hospital Centre Mohammed VI, Faculty of Medicine and Pharmacy Cadi Ayyad University Marrakesh Morocco; ^2^ Zohor anatomical pathology laboratory Cadi Ayyad University Marrakesh Morocco

**Keywords:** chemotherapy, Hodgkin's lymphoma, poor outcome, skin relapse

## Abstract

We present a case of a relapse of HL revealed by a skin involvement. A biopsy of the skin lesion showed infiltration by a mixed cellularity and Reed–Sternberg cells. The immunoreactivity was positive for CD30 and CD15. The patient was undergoing ICE protocol with good improvement after three cycles.

## Graphical abstract

We report the case of a relapse of Hodgkin's lymphoma revealed by a skin involvement characterized by a nodular scleroderma‐like placard with ulcerations at the neck, thorax, and armpits. The diagnosis should be considered in unexplained skin lesions. There is no specific treatment, and this disease responds to standard chemotherapy.

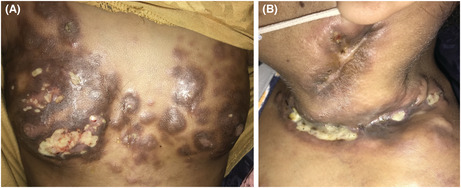

## 1 INTRODUCTION

Skin involvement in Hodgkin's lymphoma (HL) is very rare, occurring in only 0.5% to 3.5% of cases.^1^ When it occurs, it is a sign of advanced illness. The majority of patients described with skin involvement during HL were in systemic relapse with secondary skin manifestations.^2^ Its lesions may occur in 17% to 53% of patients and are usually associated with a paraneoplastic syndrome rather than cutaneous HL. Non‐specific skin involvement does not reflect skin infiltration by the tumor.^3^, ^4^, ^5^, ^6^ Skin involvement during HL is a poor prognostic sign and similar to stage IV disease.^7^, ^8^, ^9^ We report this case to describe the clinical and histological skin manifestations secondary to a systemic relapse of HL in a woman of 24 years, and their evolution under chemotherapy.

## 2 CASE PRESENTATION

**FIGURE 1:**
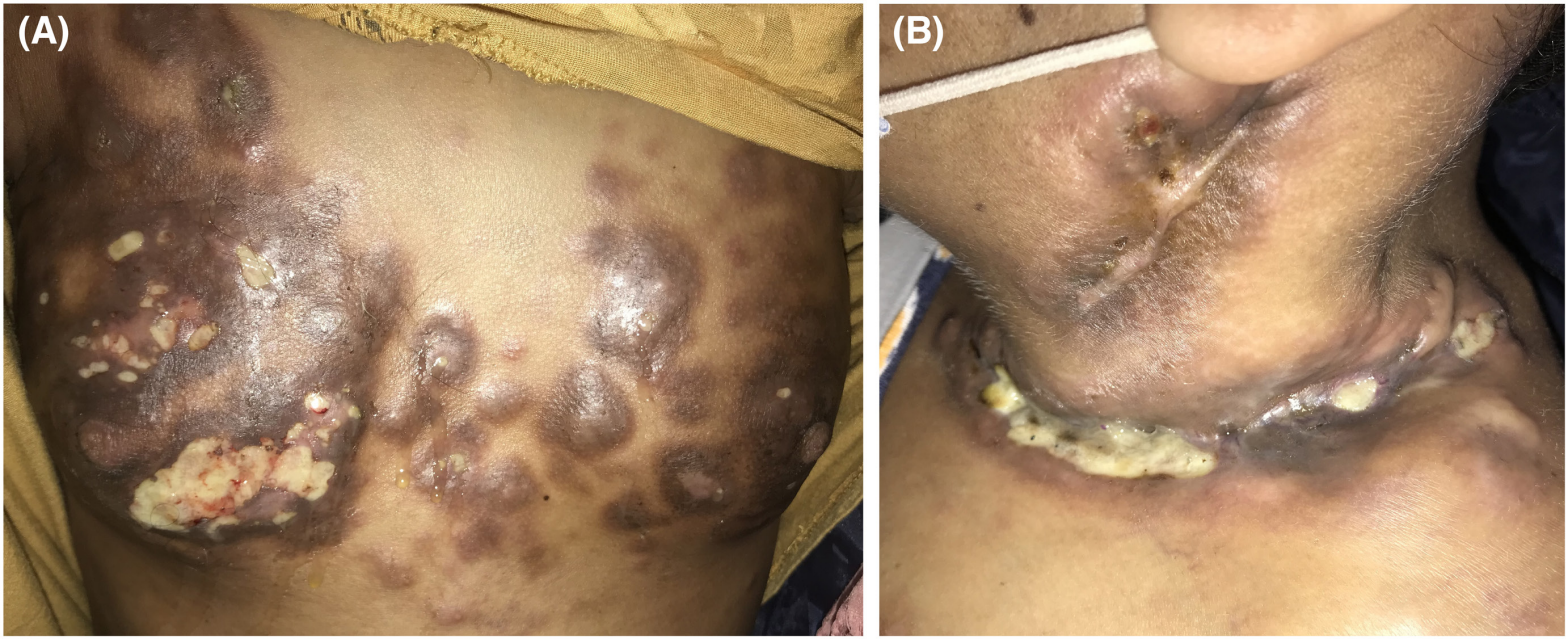
(A) Multiple nodular and ulcerated lesions on the chest wall. (B) Giant ulceration of the left basal cervical region

A 24‐year‐old woman had a five‐year history of stage IIIB Hodgkin lymphoma who received eight cycles of ABVD (Doxorubicin, bleomycin, vinblastine, and dacarbazine) with complete remission of the disease. After 5 years, the patient developed skin lesions such as a nodular sclerodermiform cupboard with giant ulcerations of the left basal cervical, two hemithoraces, and armpits (Figure 1).

**FIGURE 2:**
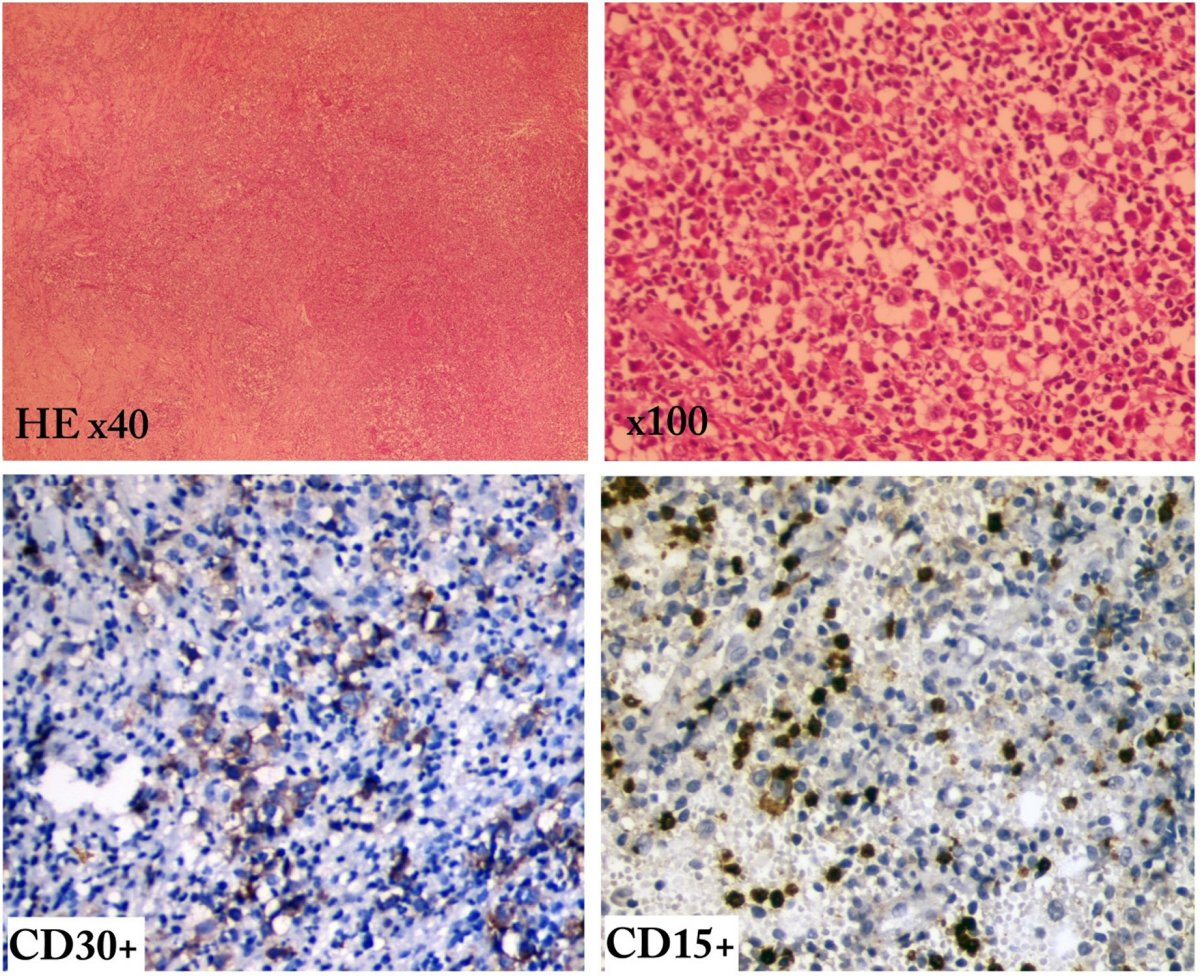
HEx40 general view with vaguely nodular infiltration of the middle and deep dermis. x100 presence of lacunar cells with eosinophilia and Reed–Sternberg cells. Anti‐CD15/CD30 positive with numerous Hodgkin cells, cytoplasmic labeling with reinforcement of Golgi

A biopsy of the skin lesion showed infiltration by a polymorphic population of large cells, accompanied by a large population of reactive inflammatory cells. It was dermal and hypodermal in location, formed by large round cells with increased nucleocytoplasmic ratio. The nuclei were round or angular with one nucleolus and dense chromatin with some mitosis. The cytoplasm was sometimes basophilic and sometimes clear. This corresponds to giant Reed–Sternberg cells. The immunoreactivity was positive for CD30 and CD15, which confirms a cutaneous and subcutaneous localization of Hodgkin lymphoma (Figure 2).

She also had fever, night sweats, weight loss, and swelling on the right side of the neck evolving for 10 months. On examination, she was pale, and the right cervical lymph nodes were enlarged, measuring 7 × 4 cm in size, firm, and non‐tender, associated with a right pleural effusion syndrome. Other systems were normal except for mild hepatomegaly.

The hematological assessment showed hemoglobin at 9 g/dl, mean cell volume at 80 fl, platelet count at 345,000/mm^3^, and white blood cells at 50,940/mm^3^, predominantly neutrophilic with 2% myelemia. Bone marrow aspiration was normal. Chest X‐ray showed a middle pleural effusion on the right side. Contrast CT scan confirmed cervical, supra, and infra diaphragmatic lymphadenopathy associated with hepatosplenomegaly and splenic nodular lesions as well as pleuropericardial and peritoneal effusion. The serum chemistry, bone marrow biopsy, and pleural fluid cytology were normal.

**FIGURE 3:**
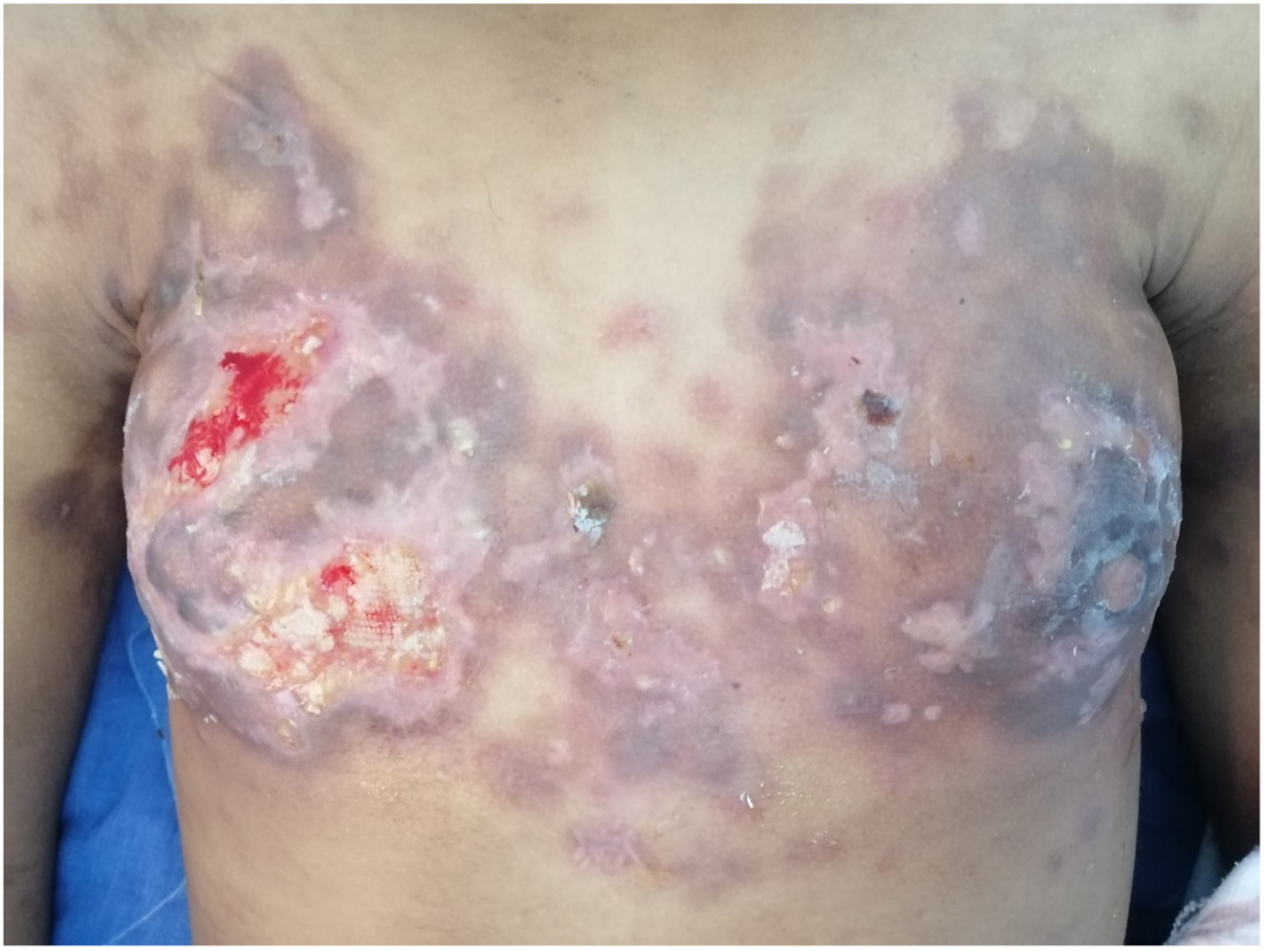
Post‐treatment photograph showing chest region with good improvement

The patient was put on an ICE protocol consisting of carboplatin (mg dose = 5 AUC) on day one, ifosfamide 5 g/m^2^/24 h on day two and etoposide 100 mg/m^2^ on days one and three, every 21 days. Good improvement of skin lesions was noted after three cycles (Figure 3). The patient died 6 months later from septic shock due to infected skin lesions.

## 3 DISCUSSION

The skin localization of HL is very rare. Its clinical presentation is heterogeneous by the presence of macules, papules, infiltrated lesions, or usually ulcerated nodules.^7^ Grosz was the first to describe HL‐specific skin lesions in 1906, observed in 0.5% to 7.5% of patients.^1^ It often occurs as a secondary manifestation, announcing a severe prognosis.^8^, ^9^ Many studies reported isolated cutaneous relapse in HL and is usually associated with systemic involvement.^2^, ^6^ This is the case with our patient, in whom the skin lesion appeared as a first sign of relapse after a long period of remission.

Unlike usual lesions, skin lesions occur in 13%–40% of patients and include pruritus, ichthyosis, hyperpigmentation, and exfoliative dermatitis.^10^, ^11^ Unusual and non‐specific skin manifestations may be observed such as lymphohistiocytic infiltrates, pyoderma gangrenosum, and atypical pityriasis rosa.^12^ Primary cutaneous HL is a distinct clinicopathologic entity morphologically and immunophenotypically from nodal HL with an indolent course in some patients.^13^ The most commonly involved areas are the trunk, the neck, and scalp.^2^ The mechanism of skin invasion, according to the hypothesis, is retrograde lymphatic extension, contiguous invasion from affected lymph nodes, suspected in our case, or hematogenous extension.^8^ Classical Reed–Sternberg cells may be difficult to locate in the skin lesion.^14^ If the diagnosis of HL has already been established by a previous lymph node biopsy, even in the absence of Reed–Sternberg cells, the diagnosis of skin lesions specific for HL should be considered.^1^ Immunohistochemistry (IHC) can be useful in difficult situations. IHC analysis of cutaneous HL has shown that CD30 was positive in both the lymph node and skin in all cases, and CD15 was positive in all lymph nodes and 70% of skin lesions.^14^ Mycosis fungoides, lymphomatoid papulosis, benign thymoma, proliferative myositis, infectious mononucleosis, and some forms of B‐cell lymphoma involving the skin, adult T‐cell lymphoma, Addison‐like areas of hyperpigmentation, prurigo, ichthyosis, herpes zoster are the differential diagnosis of cutaneous HL.^8^, ^10^, ^15^, ^16^ Lymphomatoid papulosis and anaplastic large cell lymphoma may also show CD30‐positive cells and require more specific CD15‐positive expression to be differentiated from HL.^15^ White blood cell count above 15,000/mm^3^ is a poor prognosis factor.^17^ In our patient, leukocytosis was reactive and a part of the inflammatory syndrome. The prognosis for skin HL is poor and is similar to that for patients with stage IV disease with 50% death at 1 year.^8^ There is no specific treatment for skin lesions due to HL. Cutaneous manifestations of HL respond to standard chemotherapy protocols for systemic involvement without any other therapeutic options like surgery, local therapy, and radiation.^18^ The most widely used include DHAP (dexamethasone, high‐dose cytarabine, and cisplatin) and ICE.^19^ For our patient, we adopted ICE chemotherapy with good clinical outcome. The ICE regimen followed by autologous stem cell transplantation (ASCT) is an appropriate option for relapsed HL extensively with skin involvement in terms of benefit/risk ratio with a response rate varying between 40% and 88%.^20^, ^21^, ^22^ Some studies have described a relatively benign course of skin involvement in Hodgkin lymphoma, although the prognosis remains poor.^21^ Most patients die within 1 month to 2 years from the development of skin lesions.^23^

## 4 CONCLUSION

In concluding, skin localization in HL is rare. The prognosis is commonly poor. Clinically, the diagnosis of HL should be considered in any type of unexplained skin lesions with associated lymphadenopathy. There is no specific treatment. Standard chemotherapy protocols stay the only remedy for cutaneous LH.

## AUTHOR CONTRIBUTIONS

Ouadii Abakarim involved in conceptualization, writing—original draft, and writing—revision and editing. Oumaima Maghnouj, Fatima Zahra Lahlimi, and Illias Tazi were included in the diagnosing, care, and treatment of the patient. Abderrachid Hamdaoui was the pathologist consultant. All authors approved the final version.

## FUNDING STATEMENT

This research received no specific grant from any funding agency in the public, commercial, or not‐for‐profit sectors.

## CONFLICT OF INTEREST

We have no conflicts of interest to disclose.

## CONSENT

Following the patient's death, written informed consent was obtained from the patient's brother to publish this report in accordance with the journal's patient consent policy.

## Data Availability

The data used to support the findings of this study are included within the article.
